# ST-Elevation Myocardial Infarction Due to Coronary Vasospasm Associated with Eosinophilic Granulomatosis with Polyangiitis: A Case Report

**DOI:** 10.5811/cpcem.19412

**Published:** 2024-06-14

**Authors:** Christopher Allen, Christopher Poyorena, Lauren B. Querin

**Affiliations:** * Mayo Clinic Alix School of Medicine, Scottsdale, Arizona; † Mayo Clinic Hospital Phoenix, Department of Emergency Medicine, Phoenix, Arizona

**Keywords:** *case report*, *STEMI*, *coronary artery vasospasm*, *eosinophilic granulomatosis with polyangiitis*

## Abstract

**Introduction:**

ST-elevation myocardial infarction (STEMI) can be caused by underlying coronary artery vasospasm (CAV) with or without associated atherosclerotic disease. Coronary artery vasospasm is a rare but potentially devastating manifestation of eosinophilic granulomatosis with polyangiitis (EGPA).

**Case Report:**

We describe a 54-year-old male with a known history of EGPA and coronary artery disease presenting to the emergency department with chest pain and an inferior STEMI on electrocardiogram. He was ultimately taken for coronary angiography and found to have a discrete vasospastic lesion in the right coronary artery that was treated with intra-coronary nitroglycerin and calcium channel blockers. He was continued on immunosuppressant agents (prednisone and mepolizumab) for management of EGPA and followed up with outpatient cardiology and rheumatology for vasospastic angina.

**Conclusion:**

This case highlights a rare cause of STEMI, discusses the nuances in treatment of STEMI due to CAV, and provides background on pathophysiology and treatment of EGPA.

Population Health Research Capsule What do we already know about this clinic entity?

*ST-elevation myocardial infarction (STEMI) is caused by acute plaque rupture with atherosclerotic disease. A small subset is caused by non-atherosclerotic events, including coronary artery vasospasm.*

What makes this presentation of disease reportable?

*This is a rare underlying cause of a relatively common and “can’t miss” diagnosis of STEMI in the ED.*

What is the major learning point?

*Coronary artery vasospasm (CAV) is associated with underlying vasculitis and can cause STEMI. Management should include vasodilators.*

How might this improve emergency medicine practice?

*Rare causes of STEMI, including CAV and its risk factors (eg, vasculitis), should be kept on differentials so that early intervention can be implemented.*

## INTRODUCTION

The most common cause of ST-elevation myocardial infarction (STEMI) is acute plaque rupture associated with atherosclerotic disease. However, about 5–25% of cases are caused by a non-atherosclerotic event, such as coronary artery vasospasm (CAV).^1^ Differentiating STEMI due to atherosclerosis vs CAV is challenging and often requires coronary angiography for definitive diagnosis.^1^ Although rare, CAV has been associated with vasculitis seen in rheumatologic and systemic inflammatory conditions.^2^ ^,^ ^3^ Eosinophilic granulomatosis with polyangiitis (EGPA) (formerly Churg-Strauss syndrome) is a rare, small- and medium-vessel vasculitis.^4^ Coronary artery vasospasm is a potentially devastating complication of EGPA. We report a unique case of an inferior STEMI secondary to CAV associated with underlying EGPA.

## CASE REPORT

A 54-year-old male presented to the emergency department (ED) with acute-onset, severe left-sided chest pain and diaphoresis. He had taken 81 milligrams aspirin and sublingual nitroglycerin prior to presentation, which did not alleviate his symptoms. Past medical history was notable for hypertension, hyperlipidemia, coronary artery disease (CAD), and EGPA. He had four prior right coronary artery (RCA) stents, with the most recent at an outside hospital one month prior. He also reported several months of anginal chest pain thought to be secondary to coronary vasospasm.

**Image 1.**
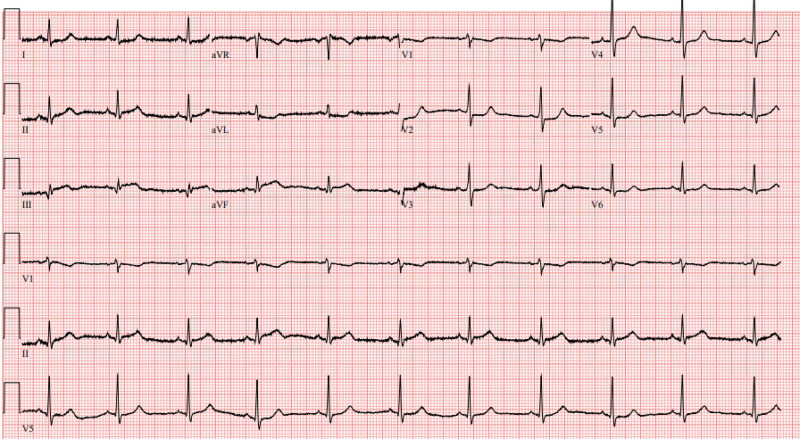
Initial electrocardiogram demonstrating a normal sinus rhythm with ST elevations in leads II, III, and aVF as well as reciprocal mild ST depressions in leads I and aVL raising concern for inferior ST-elevation myocardial infarction.

Initial vital signs included blood pressure 148/99 millimeters of mercury (mm Hg), heart rate 69 beats per minute (bpm), respiratory rate 26 breaths per minute, temperature 36.7° Celsius, and pulse oximetry 98% on room air. On exam he was diaphoretic and uncomfortable appearing with mild tachypnea, but his cardiopulmonary exam was otherwise normal. Initial electrocardiogram (ECG) (Image 1) showed a normal sinus rhythm with ST-segment changes concerning for inferior STEMI. The patient was treated with aspirin, morphine, and sublingual nitroglycerin, and a code STEMI was activated. Interval ECG obtained 15 minutes later demonstrated no significant change.

**Image 2.**
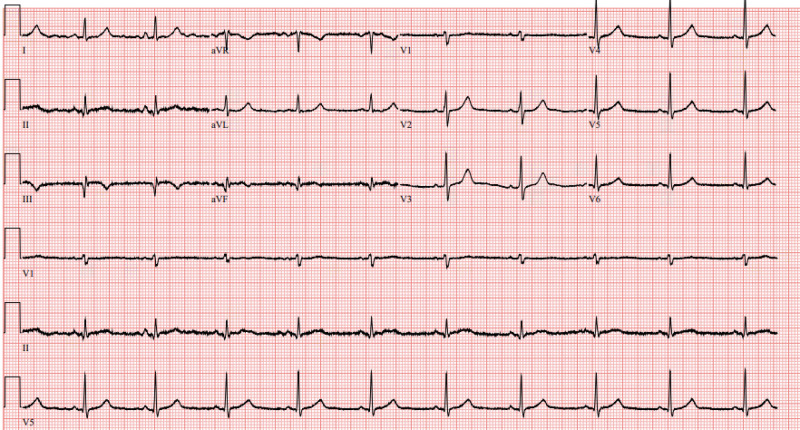
Electrocardiogram on day of discharge (post-catheterization) demonstrating a normal sinus rhythm with evidence of inferior infarct with Q waves in leads II, III, aVF and new T-wave inversions in leads III and aVF. There is resolution of previously seen ST elevations and depressions.

The patient was taken for coronary angiography, which revealed a high-grade stenosis of the distal RCA due to a discrete 99% vasospastic lesion (Figure). Intracoronary nitroglycerin was administered, ultimately resulting in resolution of the stenosis. His previous stents were patent without restenosis, and there was no significant atherosclerotic disease. Following catheterization, the patient was started on a nitroglycerin drip and diltiazem. A post-catheterization ECG (Image 2) showed evidence of recent inferior infarct. He was discharged two days later on dual antiplatelet therapy, diltiazem, isosorbide mononitrate, L-arginine, prednisone, and mepolizumab therapy.

**Figure.**
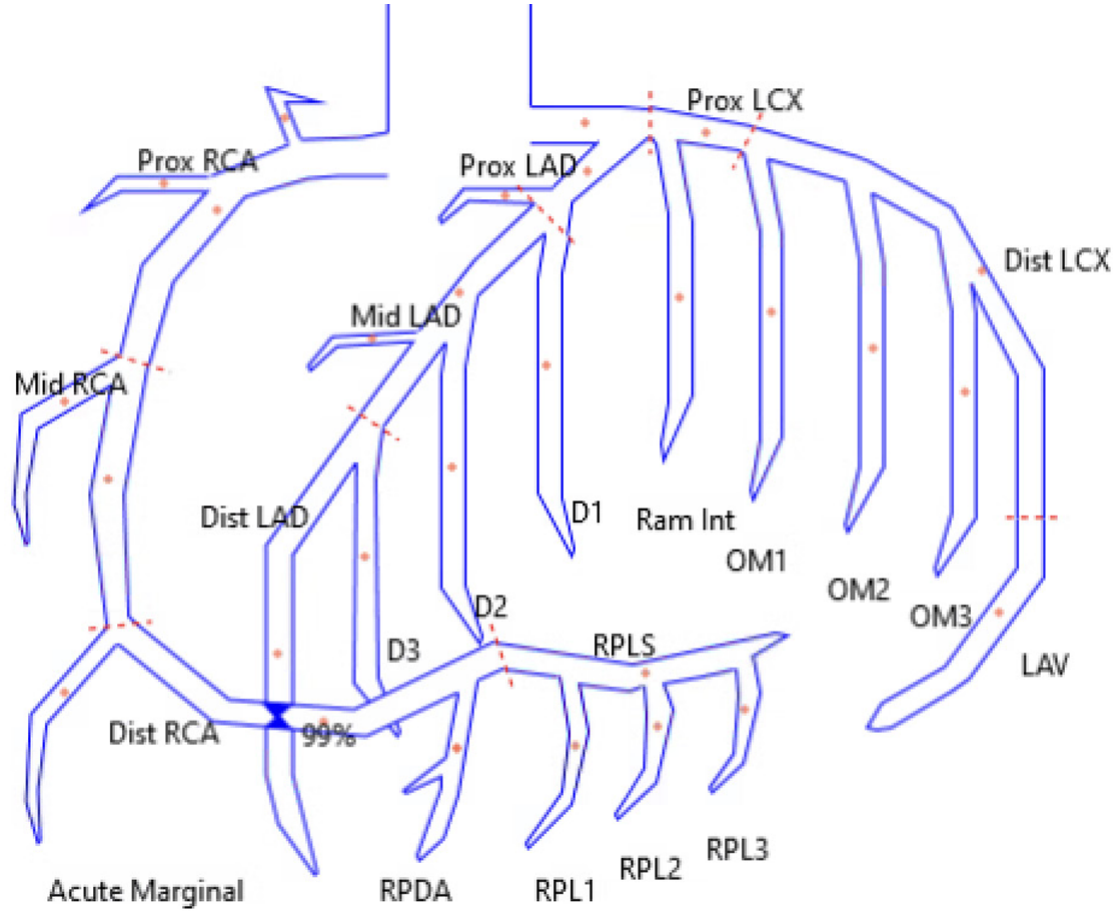
Reconstructed drawing of coronary artery anatomy demonstrating the location of the distal right coronary artery 99% stenosis. *RCA*, right coronary artery.

## DISCUSSION

Anginal chest pain secondary to CAV occurs when one or more coronary arteries constrict, leading to occlusion of the affected vessel(s). This can evolve into acute myocardial infarction (MI), as seen in this case.^1^ ^,^ ^5^ Risk factors for CAV include female gender, Japanese or Korean lineage, emotional stress, tobacco use, sympathomimetic drugs, and systemic inflammatory conditions (vasculitis).^6^ ^,^ ^7^

Eosinophilic granulomatosis with polyangiitis is a rare vasculitis thought to be caused by anti-neutrophil cytoplasmic antibody-induced endothelial damage and multisystem eosinophilic infiltration.^8^ ^,^ ^9^ Typical presenting features include adult-onset asthma, rhinosinusitis, and constitutional symptoms. However, involvement of end organs may be seen, leading to renal dysfunction, gastrointestinal symptoms, skin, and/or cardiac manifestations.^4^ ^,^ ^10^ Cardiac involvement in EGPA typically manifests as cardiomyopathy, myopericarditis, and/or dysrhythmia.^11^ Coronary artery vasospasm and MI are rarely reported. Although pathogenesis is not fully understood, it is postulated that eosinophilic inflammation induces hypercontraction of coronary smooth muscle through activation of mast cells and release of vasoactive substances.^12^ ^,^ ^13^

The presentation and initial treatment of acute vasospastic angina is the same irrespective of the underlying cause of the vasospasm. During the acute presentation of CAV, symptoms are nearly identical to atherosclerotic etiologies of acute coronary syndromes, including chest pain, diaphoresis, and dyspnea. Subtle differences exist, including that CAV often presents at rest, especially during rapid eye movement (REM) sleep and in the early morning.^5^ ^,^ ^14^ Electrocardiogram findings are variable and can range from normal to ST-elevations meeting STEMI criteria.^1^

Differentiating between atherosclerotic plaque rupture and CAV as etiology of STEMI can be challenging. However, definitive treatment differs and, therefore, physicians should be attentive in considering CAV, particularly in patients with underlying risk factors.^1^ Coronary artery vasospasm may be suggested if on repeat ECG there is resolution or improvement of ST changes following administration of fast-acting nitrate. However, definitive diagnosis requires coronary angiography, especially if the diagnosis is unclear, or if symptoms persist.^5^ Emergency physicians should initiate rapid involvement of interventional cardiology colleagues, while simultaneously starting vasodilatory treatment in patients with STEMI and suspected CAV.

Mainstay therapy of CAV is centered around vasodilation of the coronary vasculature. Non-dihydropyridine calcium channel blockers (CCB) and nitrates are the pharmacotherapies of choice.^1^ ^,^ ^14^ Additionally, lifestyle changes, treatment of underlying conditions, and optimization of cardiovascular risk, are indicated.

Primary treatment of EGPA is corticosteroids, along with other immunosuppressive agents.^15^ Treatment of vasospastic angina in EGPA is less established and dependent on acuity of presentation. Case reports in cardiology literature highlight treatment with systemic and intracoronary nitroglycerin and calcium channel blockers, as utilized in this case.^2^ ^,^ ^3^

This patient had known EGPA with a recent history of anginal chest pain that raised suspicion for vasospastic angina. However, he also had risk factors for, and previously diagnosed, CAD with a recent stent placement, which raised concern for plaque rupture or stent thrombosis. Therefore, the patient underwent coronary angiography, which allowed for definitive diagnosis and treatment. Upon discharge, the patient was continued on nitroglycerin, CCB, and dual-antiplatelet therapy. Additionally, treatment with prednisone and mepolizumab (interleukin-5 inhibitor) were continued to address the underlying EGPA in hopes of preventing recurrent vasospastic angina.

## CONCLUSION

Coronary artery vasospasm is a rare but potentially devastating manifestation of EGPA. Acute STEMI can be caused by CAV, which is primarily treated medically with nitrates and calcium channel blockers, as opposed to coronary stent placement frequently used to treat STEMI due to underlying atherosclerotic disease. Emergency physicians should maintain suspicion for CAV in patients with underlying risk factors, including in those with EGPA, in the appropriate clinical context. Initiation and referral for treatment of EGPA is also key for prevention of recurrent vasospastic angina.
